# Standardised Warfarin Reversal Expedites Time to Theatre for Fractured Neck of Femur Surgery and Improves Mortality Rates: A Matched Cohort Study

**DOI:** 10.1155/2018/4791214

**Published:** 2018-10-21

**Authors:** Thomas S. Moores, Benjamin D. Chatterton, Matthew J. Walker, Phillip J. Roberts

**Affiliations:** Department of Trauma and Orthopaedics, Royal Stoke University Hospital, Stoke-on-Trent, Staffordshire, UK

## Abstract

**Background:**

This study aims to evaluate outcomes for warfarinised hip fracture patients and compare them with a matched nonwarfarinised group, before and after the introduction of national hip fracture guidelines in the United Kingdom.

**Methods:**

A retrospective cohort study of 1743 hip fracture patients was undertaken. All patients admitted taking warfarin were identified. These patients were then matched to nonwarfarinised patients using nearest neighbour propensity score matching, accounting for age, sex, hip fracture type, and Nottingham Hip Fracture Score. A pre-guideline group (no standardised warfarin reversal regimen) and a post-guideline group (standardised regimen) were identified. Outcomes assessed included time to INR less than 1.7, time to theatre, length of stay, and 30-day and 1-year mortality.

**Results:**

Forty-six warfarinised hip fracture patients were admitted in the pre-guideline group (mean age 80.5, F:M 3:1) and 48 in the post-guideline group (mean age 81.2 years, F:M 3:1). Post-guideline patients were reversed to a safe operative INR level within 18 hours of admission, decreasing the time to first dose vitamin K (p<0.001). 70% of warfarinised patients were operated upon within 36 hours, compared to 19.6% with no regimen (p<0.05). After anticoagulation reversal protocol, thirty-day mortality decreased from 15.2% to 8.3% and 1-year mortality from 43.5% to 33% for warfarinised patients, which is comparable to nonwarfarinised matched patients. There was no significant change in the length of stay pre- and post-guideline for both groups of patients.

**Conclusions:**

Proactive anticoagulant management and expedient surgery reduces morbidity and mortality when managing this surgically challenging subset of hip fracture patients.

## 1. Introduction

### 1.1. Background and Rationale

An estimated 1% of the United Kingdom (UK) population take warfarin [1] for conditions including atrial fibrillation, venous thromboembolic disease (deep vein thrombosis or pulmonary embolism), cerebrovascular disease, or following cardiac valve surgery [2]. It is estimated that 3.2% of hip fracture patients are admitted taking warfarin [3]; this figure is anticipated to increase in accordance with the predicted rising hip fracture incidence [4] in the UK. There are an estimated 80,000 hip fracture cases per year in the UK [5], and therefore approximately 2500 hip fracture patients will be admitted taking warfarin each year [3]. Traditionally, these patients were considered at high risk, due to the increased risk of bleeding and associated perioperative complications. This subset of hip fracture patients poses a surgical challenge, with UK National Institute of Health and Care Excellence (NICE) guidance [4] focusing upon holistic multidisciplinary patient care, including safely optimising anticoagulation and other comorbidities to undergo emergency surgery [4]. This strategy decreases morbidity and mortality for all hip fracture patients [6–8]. Although no agreed national guidelines exist for the reversal of warfarin in the UK, the use of standardised reversal regimens has been shown to be beneficial in-patient optimisation and reduces their delay to theatre [9, 10]. This, combined with national incentivised “Best Practice” tariff (BPT) [11] and NICE guidelines on holistic care for hip fracture patients [4], has reduced mortality and morbidity [12, 13].

Warfarin is a vitamin K antagonist derived from coumarin [14]. Options to reverse its effect include oral vitamin K, intravenous vitamin K at either high or low dosages, dose omission, and blood products, including fresh frozen plasma (FFP) or prothrombin complex concentrates (PCC) [9, 10, 15–17]. Oral vitamin K takes up to 48 hours to be effective. Blood products have a limited window of effectiveness, need to be used in conjunction with vitamin K, and have additional problems of cost and adverse reactions [9, 10]. Intravenous vitamin K is advocated if surgery can be delayed beyond 12 hours; otherwise blood products may have a role [18–20] to reach a safe operative INR [21, 22]. Seventy-seven percent of delays to theatre beyond 48 hours in hip fracture patients are the result of poorly controlled medical comorbidity or pending investigations [23, 24]. Anticoagulation reversal accounts for 11% of comorbidity related delays [24], which is associated with increased 30-day mortality [4, 7, 25, 26]. Outcomes for warfarinised patients have been recently reported by Lawrence et al., showing that after controlling for prognostic factors such as age, ASA, and AMTS, warfarin therapy at the time of hip fracture is associated with increased time to surgery, length of stay, and increased mortality [27]. However, there has been no analysis against a matched group of patients who were not anticoagulated with warfarin on admission.

### 1.2. Objectives

We hypothesised that the introduction of our standardised regimen for the reversal of anticoagulation in fractured neck of femur patients would expedite their surgery and possibly reduce subsequent length of stay and post-operative mortality. The aims of this study were to evaluate outcomes for warfarinised patients admitted with a hip fracture before and after the introduction of a standardised reversal regimen for warfarin, compared to matched nonwarfarinised patients. The measurable outcomes chosen were time to theatre, length of stay, and post-operative mortality.

## 2. Patients and Methods

### 2.1. Study Design

This study was a retrospective analysis of prospectively collected matched data. A search through our institution's database was performed on all patients admitted to our institution between the 1^st^ of January 2008 and 28^th^ February 2012 with a hip fracture. A list of patients admitted between these dates for whom the primary International Classification of Diseases-10 (ICD-10) diagnosis code for their spell corresponded to hip fracture (S72.0, fracture of neck of femur; S72.1, pertrochanteric fracture; S72.2, subtrochanteric fracture) was generated [28]. These patients were cross-referenced with our institutions admissions database to ensure that the data were accurate. This was further cross-referenced with our institutions National Hip Fracture Database (NHFD) records for all hip fracture patients input onto the National Audit Programme [12]. No patients were excluded. The key outcomes assessed included time to first dose of vitamin K and International Normalized Ratio (INR) less than 1.7 (this is our centres agreed safe operative level by surgeons and anaesthetists), time to theatre, length of stay, and 30-day and 1-year mortality. The time to INR of less than 1.7 is taken from time of admission to the time of publication of the laboratory published results on the electronic pathology system for all cases.

### 2.2. Identification of Patients

From the generated hip fracture patient list, comorbidities were recorded on our institutions' admissions database, which allowed us to identify all patients admitted on warfarin for any reason. These patients were assigned to the “warfarinised” arm of the study. This cohort continued with their warfarin therapy long-term following surgery. The generated hip fracture patient list was also the source of the matched nonwarfarinised patients. This cohort were assigned to the “matched” arm of the study. Post-operatively, the matched cohort were given the standard prophylactic regimen of 4 weeks daily subcutaneous dalteparin as venous thromboembolic event (VTE) prophylaxis.

Warfarinised patients were divided into a pre-guideline and post-guideline group, with the introduction of the BPT of April 2010 being the delineation between these groups. The pre-guideline group included all warfarinised patients between 1^st^ January 2008 and 31^st^ March 2010, whilst the post-guideline group included all patients between 1^st^ April 2010 and 28^th^ February 2012. The pre-guideline group were not treated with a standardised warfarin reversal protocol and received variable amounts of intravenous vitamin K, at variable times based on the clinical judgement of the clinical team. The post-guideline group received a standardised protocol of low dose 2mg intravenous vitamin K every 6 hours from admission until their INR was less than 1.7.

Electronic patient records were used to establish patient demographics, Nottingham Hip Fracture Score, admission date, time to theatre, discharge date, and date of death for each patient, allowing calculation of 30-day and 1-year mortality. The fracture pattern was obtained from the patient's ICD-10 primary diagnosis code [28]. These were categorised as (i) intracapsular fracture and (ii) extracapsular and subtrochanteric fractures and cross-referenced with the admission database and PACS (Picture Archiving and Communication System) radiological software. Theatre and anaesthetic databases were accessed for timings to theatre, operation type, and ASA grade. The time to theatre was calculated from time of admission in the Emergency Department to the time the patient entered the anaesthetic room in hours. Timings to theatre were categorised as (i) less than 36 hours, (ii) 36-48 hours, (iii) greater than 48 hours, and (iv) no operation. These categories have been devised because the BPT target for time to theatre is less than 36 hours, and the NICE target is less than 48 hours, allowing comparison of our data with the National Hip Fracture Database (NHFD). Length of stay was expressed in days and is the time from admission in A&E to discharge from orthopaedic care and relates to their acute hospital spell. Thirty-day mortality was calculated by including patients who had died on any day up to and including day 30 following admission, and for 1-year mortality calculation patients who died up to and including 365 days from their day of admission were included in the analysis.

### 2.3. Statistical Analysis

#### 2.3.1. Patient Matching

The pre-guideline and post-guideline warfarinised cohorts were matched separately with nonwarfarinised patients using a “one to one nearest neighbour model” of propensity score matching for the variables of age (years), sex, fracture type, and Nottingham Hip Fracture Score [29]. Patient matching was necessary to compare outcomes for patients admitted on warfarin versus those who were not anticoagulated. Once each warfarin case had been matched to a nonwarfarin case, that patient was eliminated from any further matching to avoid duplication of patients and allow direct comparison of outcomes for these patients.

#### 2.3.2. Outcomes

Data were collated in Microsoft Excel 2010, and data analysis was performed using SPSS Statistics for Windows (version 22.0, IBM Corp., Armonk, NY, 2013) and the statistical package R (R version 3.0.2 (2012-09-25)) [30]. Descriptive statistics were used to describe the study population, with independent samples t-tests used to analyse continuous variables and chi-squared test used to analyse categorical variables. Statistical significance was assumed if p<0.05. For the pre-guideline and post-guideline groups their matched nonwarfarinised cases were compared for any difference in age, sex, fracture type, and Nottingham Hip Fracture Score (NHFS). Kaplan-Meier survival curves were used to compare survival between warfarinised and nonwarfarinised patients in the pre-guideline and post-guideline groups. Additionally, a further analysis of survival comparing warfarinised patients from the pre-guideline group to warfarinised patients in the post-guideline group was undertaken, with patient death being the survival outcome in both cases. This was calculated from day of admission to date of death or date of final follow-up. Chi-squared analysis was used to compare the survival curves between the two groups, with a p value of less than 0.05 (p<0.05) being considered significant.

## 3. Results

### 3.1. Study Population

A total of 1743 patients were initially identified from electronic patient records, 786 from the pre-guideline group and 957 from the post-guideline group. In the pre-guideline group 46 patients (5.9%) were admitted taking warfarin (33 females, 13 males), with a mean age of 80.5 years (±7.6 years, range 61-93 years). There were 26 intracapsular hip fractures and 20 extracapsular hip fractures, and the mean NHFS predicted a 9.2% (±6.5%) 30-day operative mortality. In the post-guideline group 48 patients (5.0%) were admitted taking warfarin (32 females, 16 males), with a mean age of 81.2 years (±8.0 years, range 62-100 years). There were 28 intracapsular hip fractures and 20 extracapsular hip fractures, and the mean NHFS predicted a 7.2% (±3.7%) 30-day operative mortality. Each patient was matched for age, sex, fracture type, and NHFS; there was no significant difference in these variables between warfarinised or nonwarfarinised patients in the pre-guideline group. In the post-guideline group there was no statistical difference between variables except in NHFS. The nonwarfarinised matched group had a mean NHFS of 9.8% (±7.6%) for 30-day operative mortality; this was statistically significant compared to the mean 7.2% for the warfarinised controls (p=0.04). This data is summarised in Table 1.

### 3.2. Comparison of Warfarinised Groups Pre- and Post-Guideline

Comparing the demographics of the two warfarinised groups showed no statistical difference in age (p=0.641), in sex 3:1 female (p=0.901) to male (p=0.743) ratio, or between extracapsular (p=1.000) and intracapsular (p=0.960) fractures. There was a statistically significant difference (p=0.006) between mean NHFS of 9.2 (±6.5) and 7.2 (±3.7), for pre-guideline and post-guideline groups, respectively.

### 3.3. Warfarin Reversal Management to Safe Operative Level

As shown in Table 1, the mean admission INR was 2.6 and 2.5 for the pre-guideline and post-guideline warfarinised groups, respectively. In the pre-guideline group there was no standardised reversal regimen, and each patient received different treatment. Patients received either no treatment or intravenous vitamin K, with doses ranging from 1mg to 10mg. The mean time from admission to administering the first dose of intravenous vitamin K was 13.07 hours (± 7.07), with a range of 1-28 hours after admission.

In the post-guideline group all patients received the same standardised reversal regimen with a mean time to 1^st^ dose vitamin K of 3 hours; comparing this to the pre-guideline group of 13.07 hours, this result was statistically significant (please see Table 2).

### 3.4. Time to Theatre

As shown in Table 3, there was a mean delay to theatre of 70.36 hours (± 61.9 hours) from admission for the warfarinised patients, pre-guideline. The mean time to theatre for the matched pre-guideline nonwarfarinised patients was significantly lower, 44 hours (p=0.03). In the post-guideline patients who were on warfarin, there was a mean time to theatre of 46 hours (± 37.927 hours). The mean time to theatre for those not taking warfarin was 43 hours (p=0.755).

In the pre-guideline group of patients taking warfarin, 19.6% were operated upon in less than 36 hours compared to 45.7% of the nonwarfarinised group at the same time. Following the introduction of a local warfarin reversal protocol and incentivised guidelines, 70.8% of patients taking warfarin were operated upon within 36 hours, which is comparable to the 77% of nonwarfarinised patients at the same time. Prior to the introduction of national guidelines giving the 36 [18] and 48-hour [19] targets, there were no specific targets, but the NHFD reported outcomes for surgery within 48 hours [26]. Therefore, comparing the pre-guideline groups, 28.3% of warfarinised patients and 65.2% of nonwarfarinised patients were operated upon within 48 hours. After national targets were introduced, these figures improved to 77.1% of warfarinised patients and 85.4% of nonwarfarinised patients having surgery within the 48-hour target. Since the introduction of a local warfarin reversal protocol there has been a statistically significant decrease in time to theatre for 36 hours (p=0.039) and 48 hours (p=0.041) when comparing the two warfarinised groups.

### 3.5. Length of Stay

The mean length of stay was 5.1 and 1.6 days less for the nonwarfarinised matched patients in the pre- and post-guideline groups, respectively (p=0.10 and 0.22). Following the introduction of the local warfarin protocol, the length of stay increased by 5 days; however, this was not statistically significant (p=0.408) (Table 4).

### 3.6. Mortality

**Figure 1:**
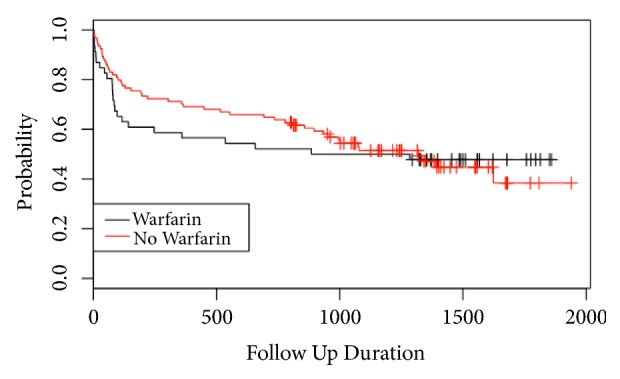
Kaplan-Meier curve of pre-guideline matched warfarinised and nonwarfarinised patients during 54-month follow-up (follow-up duration in days).

Referring to Table 5, in the pre-guideline group, 30-day mortality for the warfarinised and nonwarfarinised patients was 15.2% and 8.7%, respectively (p=0.34). One-year mortality was 43.5% and 26.1% for the warfarinised and matched nonwarfarinised patients, respectively (p=0.15). There was a mean follow-up of 54 months allowing Kaplan-Meier analysis and chi-squared analysis of the difference between the two curves (see Figure 1).

**Figure 2:**
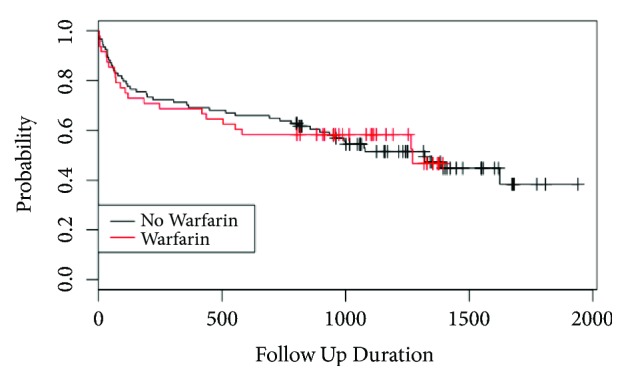
Kaplan-Meier curve of post-guideline matched warfarinised and nonwarfarinised patients during 58-month follow-up (follow-up duration in days).

In the post-guideline group, 30-day mortality for the warfarinised and nonwarfarinised patients was 8.3% and 6.2%, respectively (p=0.63). One-year mortality was 33.3% and 33.3%, respectively, for the warfarinised and matched nonwarfarinised patients (p=1.00). There was a mean follow-up of 38 months allowing Kaplan-Meier analysis and chi-squared analysis of the difference between the two curves (see Figure 2). There was no statistical difference in long-term survival between warfarinised and nonwarfarinised patients (p=0.992). There was also no significant difference in long-term survival between pre- and post-guideline warfarinised patients (p=0.67) and between pre- and post-guideline matched patients (p=0.43)

## 4. Discussion

The NHFD has shown that introduction of national guidelines has had a significant beneficial effect on outcomes for all hip fracture patients [12]. However, they do not identify specific subsets of high-risk patients who may benefit from specific optimisation, for example, warfarinised patients requiring an anticoagulation reversal protocol to ensure national targets are met. Patients admitted with a hip fracture requiring emergency surgery whilst being anticoagulated with warfarin pose a challenging surgical dilemma. Our findings suggest that having a warfarin reversal protocol as part of preoperative optimisation has significantly reduced the time to theatre for this challenging subset of patients. The majority of warfarinised patients (77%) are undergoing their emergency surgery within the national targets, which has likely contributed to a reduced length of stay and decreased 30-day and 1-year mortality [4, 11].

Reviewing the NHFD reveals that the average age for hip fracture patients is 80 years, with a 3:1 female to male ratio [4]. Our pre-guideline cases had a mean age of 80.5 with 71.7% being female, which is consistent with the NHFD audit demographic data. In the post-guideline group the mean age was slightly higher at 81.2 years, with 33% of patients being male; this differs from the NHFD population, with males known to have poorer outcomes following hip fracture [31]. The national average life expectancy in 2010 was 79 years for males and 83 years for females [32], with our cohort having their hip fracture at approximately this age and the age range for the cohorts reaching 93 years and 100 years in the pre- and post-guideline groups, respectively. These patients would have a high mortality risk due to their age and comorbidities even prior to the additional increase in mortality risk associated with hip fracture, which is an independent clinical risk factor [33]. A comparable distribution is seen when comparing the type of fracture to that recorded in the NHFD (57% intracapsular types, 40% extracapsular types, and 3% unknown) [12].

The previously reported proportion of patients taking warfarin admitted with a hip fracture is reported as a minimum of 3.2% [3]. This was higher in our study population, being 5.9% and 5.0% for pre- and post-guideline groups, respectively. As patients live longer, they have an increased risk of developing comorbidities requiring warfarin therapy to reduce their embolic relative risk [3, 17]. Our warfarinised patients were older than those seen in Ashouri et al.'s population, which may explain our higher proportion of warfarinised patients [3].

The biggest delay in warfarin reversal was the time to the first dose of vitamin K. Without a protocol this was poorly addressed with a mean time to first dose of 13 hours. This significantly reduced to 3 hours (p<0.001) after protocol introduction, with all patients INR being in a safe operative range within 24 hours of admission. It is well reported that the early hip fracture surgery is associated with improved outcomes, with national guidance suggesting surgery within 48 hours [4, 7, 8, 11]. Indeed, recent studies have suggested that outcomes deteriorate when surgery is delayed beyond 12 hours [6]. Repeated low dose intravenous vitamin K is appropriate to achieve surgery within 36 hours [9], but if surgery within 12 hours was necessary to improve outcomes, then blood products would need to be considered [18]. The use of FFP and PCC is unreported in hip fracture literature and should be given with caution and on haematology advice, due to potential complications including Transfusion Related Acute Lung Injury (TRALI), anaphylaxis, blood borne infections (HIV and Hepatitis B), and allo-immunisation [34, 35].

Length of stay has fallen since the introduction of the national guidelines but was greater for warfarinised patients compared to their matched controls in both the pre- and post-guideline groups. In the pre-guideline group the delay in preoperative reversal of warfarin contributed, with delayed surgery being associated with increased length of stay, morbidity including pressure sores and respiratory infections, and mortality [7], to complications not seen in our cohort.

The NHFD reports on 30-day and 1-year mortality, but there is no long-term mortality data. For our cohorts we have mortality data reported at final follow-up of mean 54 months and 34 months in the pre- and post-guideline groups, respectively. Mortality at 30 days and 1 year was higher in the pre-guideline warfarinised group (15.2% and 43.5%) compared to their matched cases (8.7% and 26.1%). This supports the proposition that delays to theatre and poor preoperative warfarin anticoagulation reversal are associated with poorer outcomes in warfarinised patients compared to their matched cases [7, 8, 31]. Chatterton et al. reported post-mortem data for deaths within 30 days with pneumonia being the commonest cause of death [13]. Given the delays to theatre preoperatively (mean 70 hours), warfarinised patients were at high risk for pneumonia secondary to poor lung ventilation because of immobility, posture, opiate medications, and pain. Following the introduction of the warfarin reversal protocol and national incentivised guidelines, 30-day mortality for the warfarinised and nonwarfarinised patients had reduced to 8.3% and 6.2%, respectively (p=0.63), which is comparable to the NHFD data [12]. Reversing the INR rapidly and safely has meant that 77% of patients were operated upon within 48 hours, which is known to improve outcomes [7, 8, 32, 36]. One-year mortality was 33.3% in both the warfarinised and matched nonwarfarinised patients (Figure 2), with no difference between mortality curves (p=0.992). Long-term follow-up is rare for hip fracture patients, and the Kaplan-Meier curves demonstrate that mortality is greatest in the first year followed by mortality plateaus with no difference (pre-guideline p=0.776, post-guideline p=0.992) between the warfarinised and nonwarfarinised curves by 54 and 38 months of follow-up, respectively.

The strengths of this study design include the case matching to enable comparison between outcomes in warfarinised and nonwarfarinised hip fracture patients. Matching was utilised to address the issues of confounding in the design phase of the study. The method of matching used a one to one nearest neighbour model [29], which allows direct analysis of treatment of anticoagulation reversal compared to those not anticoagulated at admission. Limitations include the patient list being generated utilising the ICD-10 codes for hip fracture [28]. This should not have affected our study because hip fracture coding in the UK has not changed since 1995 [37], predating our study period. In addition, a systematic review of 32 studies has shown that discharge coding accuracy levels are sufficiently robust for research use [38]. Furthermore, our data was cross-referenced with multiple sources to confirm accuracy. Due to the retrospective nature of this study we were also unable to obtain specific data on post-operative complications including wound issues, thrombotic events, or pressure sores.

This matched cohort study of a surgically challenging subset of hip fracture patients shows poorer outcomes for warfarinised patients than a matched cohort of nonwarfarinised patients. Implementing a proactive warfarin reversal policy coupled with national incentivised care standards facilitates rapid surgery, improving holistic care and 30-day and 1-year mortality. Intravenous vitamin K is safe to achieve surgery within 36 hours; however if surgery within 12 hours could improve outcomes further, then blood products may be required, an area that requires further research.

## Figures and Tables

**Table 1:** Summary of study population demographics for propensity score matched cohorts pre- and post-guidelines. (M: male, F: female, IC: intracapsular, EC: extracapsular; comparing warfarin cases column relates to the warfarin cases pre- and post-guideline; *∗* p < 0.05 is statistically significant.)

		Pre-Guideline			Post-guideline			P-Value
		Case	Matched	P-value	Case	Matched	P-value	Comparing warfarin cases
Total		46	46/786		48	48/957		

Age years	Mean	80.5	80.5	0.97	81.2	81.3	0.97	0.641
		(±7.6)	(+/- 7.4)		(+/- 8.0)	(+/- 8.3)		
	Range	61-93	61-93		62-100	62-104		

Sex (%)	M	13 (28.3)	14 (30.4)	0.89	16 (33.3)	17 (35.4)	0.92	0.743
	F	33 (71.7)	32 (69.6)	0.93	32 (66.7)	31 (64.6)	0.9	0.901

NHFS		9.2	8.9	0.86	7.2	9.8	$\underline{*}$ 0.04	$\underline{*}$ 0.006
					(+/- 3.7)	(+/- 7.6)		
		(+/- 6.5)	(+/- 5.2)					

Fracture Type (%)	IC	26 (57)	26 (57)	1.0	28 (58)	28 (58)	1.0	0.960
	EC	20 (43)	20 (43)	1.0	20 (42)	20 (42)	1.0	1.0

INR on Admission	Mean	2.6 (+/-0.9)			2.5 (+/- 1.0)			0.87
	Range	1.2-5.1			1.4-4.9			

**Table 2:** A comparison of pre- and post-guideline time to first dose of 2mg intravenous vitamin K from admission.

		Pre-Guideline	Post-Guideline	P Value
Time to 1^st^ dose Vitamin K (Hours)	Mean	13.07	3.00	$\underline{*}$ <0.001
		(+/-7.07)	(+/-1.11)	
	Range			
		1-28	2-6	

**Table 3:** A comparison of time to theatre between pre- and post-guideline, and warfarinised and matched patients.

		Pre-Guideline			Post-Guideline			Odds Ratio (95% CI) Pre- and Post-Guideline		P Value
		Case	Matched	P-value	Case	Matched	P-value	Comparing Warfarin Cases	Comparing Matched Cases	Comparing warfarin cases
Time to Theatre (Hours)	Mean	70.4	44.2	$\underline{*}$ 0.039	46	42.5	0.79			$\underline{*}$ 0.036
	(SD)	(61.93)	(40.97)		(37.97(	(80.90)				

	Range	13-307	9-183		12-183	13-571				

Time to Theatre (Hours) n (%)	<36	9 (19.6)	21 (45.7)	$\underline{*}$ 0.024	34 (70.8)	37 (77.1)	0.279	7.83	4.50	$\underline{*}$ 0.039
								(2.96-20.70)	(1.76-11.51)	
	36-48	4 (8.7)	9 (19.6)		3 (6.3)	4 (8.3)				$\underline{*}$ 0.041
	<48	13 (28.3)	30 (28.3)	$\underline{*}$ 0.002	37	41 (85.4)	0.12	6.47	3.83	<0.001
								(2.50-16.72)	(1.24-11.78)	
	>48	25 (54.3)	14 (30.4)		11 (22.9)	5 (10.4)				$\underline{*}$ 0.047
	No op	8 (17.4)	2 (4.3)		0 (0)	2 (4.2)				

**Table 4:** A comparison of pre- and post-guideline length of stay between warfarinised and nonwarfarinised matched patients.

		Pre-Guideline			Post-Guideline			Change in LOS		P Value	
		Case	Matched	P Value	Case	Matched	P Value	Case	Matched	Comparing Warfarin Cases	Comparing Matched Cases
Length of Stay (Days)	Mean	17.3	13.2	0.10	22.4	14.8	0.22	5.1	1.6	0.408	0.53
	(SD)	(14.2)	(9.4)		(39.7)	(15.1)					

**Table 5:** A comparison of pre- and post-guideline mortality % at 30 days and at 1 year for warfarinised patients and nonwarfarinised matched patients.

		Pre-Guideline				Post-Guideline				Hazard Ratio (95% CI)		P Value
		Case	Matched	P Value	Hazard Ratio (95% CI)	Case	Matched	P Value	Hazard Ratio (95% CI)	Comparing Warfarin Cases	Comparing Matched Cases	Comparing Warfarin Cases
Mortality (%)	30-Day	15.2	8.7	0.34	1.81 (0.14-5.90)	8.3	6.2	0.63	1.36 (0.44-4.16)	0.97 (0.48-1.96)	1.42 (0.76-1.96)	0.299
	1 Year	43.5	26.1	0.15	1.70 (1.34-2.15)	33.3	33.3	1.0	0.96 (0.75-1.24)	1.16 (0.65-2.07)	0.86 (0.41-1.78)	0.312

## Data Availability

All data collected for the construction of this research paper are available on the database of the Royal Stoke University Hospital fractured neck of femur database, which is overseen by the senior author of this paper. The collected and processed data used for the statistical calculations are on a spreadsheet in the possession of all authors. Both the database and spreadsheet can be accessed on request.
